# The availability of priority medicines for children under 5 years in eThekwini, South Africa

**DOI:** 10.1186/s40545-021-00402-y

**Published:** 2022-01-05

**Authors:** Shannice Mahadeo, Keshmika Narain, Lungelo Mhlongo, Desmaine Chetty, Lindelani Masondo, Mandla Zungu, Fatima Suleman, Velisha Ann Perumal-Pillay

**Affiliations:** Discipline of Pharmaceutical Sciences, University of KwaZulu-Natal, Private Bag X54001, Durban, 4000 South Africa

**Keywords:** Essential medicines lists, Priority medicines, Children under 5 years, Availability, Affordability

## Abstract

**Background:**

Globally, an estimated 8.1 million children under 5 years die annually in developing countries. Ensuring essential medicines are accessible and affordable to the population is key to saving lives. This study investigated accessibility, availability and affordability of a basket of priority medicines for children under 5 years in public and private healthcare sector pharmacies in the eThekwini Metropolitan area in Durban, South Africa.

**Methods:**

The WHO/HAI survey tool for assessing medicine prices, availability and affordability was adapted and employed for a basket of WHO Priority life-saving medicines for children under 5 years. Six district hospitals in the north, south and central eThekwini Metropolitan were selected as major facility reference points and for data collection and pharmacies within a 5 km radius from each major facility were also invited to participate in the study, as outlined in the WHO/HAI tool methodology. Of the 58 pharmacies selected, a total of 27 pharmacies from both private and public healthcare sectors agreed to participate and were surveyed, representing a 47% response rate. Data was analysed using Microsoft excel.

**Results:**

All participating pharmacies (and hence the selected basket of priority medicines at these facilities) were deemed accessible. Overall the public sector had more medicines available on the shelf (averaging 64%) than the private sector (48%) which had more medicines available on order (84%). At least one medicine for each of the eight (8) conditions was available at both sectors which meant patients could be treated for these conditions. Medicines for priority conditions (except HIV, which was a 28-day course) were deemed affordable as these regimens were obtainable within a day’s wage for the lowest paid unskilled worker. Priority medicines for children under 5 years were more available and more affordable in the public sector.

**Conclusion:**

The basket of WHO essential medicines for priority conditions for children under 5 years were accessible, available and affordable in the eThekwini Metropolitan areas. This was the first study in eThekwini to determine access to the WHO basket of priority medicines for children and can be scaled-up to a national study to provide a holistic comparison of these medicines in the country, and also for global comparison.

**Supplementary Information:**

The online version contains supplementary material available at 10.1186/s40545-021-00402-y.

## Background

Access to medicines means “having medicines continuously available and affordable at public or private health facilities or medicine outlets that are within 1 h walk of the population” [1]. Access to medicines is crucial in the fulfilment of the human right to the highest attainable standard of health [2], yet globally millions of people go without essential life-saving treatments due to numerous barriers. Major factors that impede access to treatment in low–middle income countries include poor availability of medicines and high medicine prices leading to poor affordability [3, 4]. Poor access to medicines is a major public health issue as disease progression and death can be prevented if the correct medicines are made available at affordable prices to those in need.

Improving access to safe and effective medicines is one of the United Nations Sustainable Development Goals. Numerous countries, globally, subscribe to these goals as they move towards achieving Universal Health Coverage (UHC) which requires adequate access to safe, effective, affordable and quality medicines and vaccines [1, 5]. South Africa is also moving towards UHC through the National Health Insurance (NHI) financing system [6] which is intended to address the healthcare inequities inherited from the past apartheid system. According to the World Health Organisation (WHO), a well-functioning healthcare system ensures equitable access to essential medicines, vaccines and technologies that are of assured quality, safety, and efficacy and which are cost-effective [7].

South Africa has a very high neonatal mortality rate (Child mortality: 12/1000 live births,) [8]. Ensuring access to priority medicines is a means to prevent child mortality. Priority medicines for children under 5 are those medicines used to treat conditions that the World Health Organization (WHO) suggests can be prevented or treated with access to simple and affordable medicines [9]. It is, therefore, important to evaluate access to these priority medicines to gauge if the South African healthcare system (both public and private sectors) is catering to the population needs for these priority conditions for children outlined in the 2012 WHO list of priority life-saving medicines for women and children [9].

This study, therefore, investigated accessibility, availability and affordability of a basket of priority medicines for children under 5 years in public and private healthcare sector pharmacies in the eThekwini Metropolitan area in Durban, South Africa.

## Methods

### Study design

This was a quantitative, cross-sectional study, conducted from August 2018 to September 2018, in eThekwini, KwaZulu-Natal, South Africa.

### Study setting

KwaZulu-Natal is one of South Africa’s nine provinces, which has the highest birth occurrences compared to other provinces, with eThekwini being the only metropolitan municipality with ten district municipalities. The eThekwini Metropolitan area was selected for this study as the largest population resided in the central urban core [approximately 1.18 million people (34.54%), followed by the northern region (1.15 million (33.16%)], southern region [758,000 (22.03%)] and the rural/peri-urban which was 338,000 people (9.82%). Hence, the study was conducted in the more densely populated urban area [10]. The country operates as a District-based healthcare system, leaving the peoples’ care in the hands of district management. Patients within eThekwini receive medicines from 233 Primary Healthcare (PHC) facilities [11], 8 Community Healthcare centres (CHC), 17 District Hospitals [12] and Private sector pharmacies. This study evaluated the availability, affordability and accessibility of medicines in both public and private sectors of healthcare in eThekwini, using the WHO recommended list of priority life-saving medicines for children. Private sector hospital pharmacies were excluded.

### Site selection

The method for selecting data collection sites was based on the WHO/HAI survey tool for assessing medicine prices, availability and affordability [13]. Six district hospitals in the north, south and central eThekwini Metropolitan were identified as major facility reference points and as sites for data collection. Pharmacies (public and/or private sector) within a 5 km radius from each major facility were selected and invited to participate in the study. This meant 42 private sector pharmacies and 16 public sector facilities (3 PHC facilities (as a pilot for the first level of care); 7 CHC and 6 district hospital facilities) were selected and invited to participate. Of the 58 pharmacies selected, a total of 27 pharmacies from both private (*n* = 14) and public (*n* = 13) healthcare sectors agreed to participate and were surveyed. This represented a 47% response rate.

### Data collection

The internationally validated WHO/HAI Tool [13] was adapted and utilized for data collection. This study assessed three components for medicines access: (1) accessibility (defined as “having medicines continuously available and affordable at public/private healthcare facilities that are within 1 h walk of the population”) [1]; (2) availability (conducted according to WHO recommended strengths and dosage forms stipulated on the 2012 priority list of life-saving medicines for women and children [9] for 8 conditions and 56 medicines, including vaccines for children under 5 years). Medicines that were not available on the shelf on the day of data collection but available on order were dealt with separately and regarded as not available as patients would need to source the medicines from another pharmacy if treatment was required immediately); and (3) affordability (the single exit price (SEP), dispensing fee and other additional fees were used to calculate the final price a patient would pay for a specific regimen at a private sector pharmacy and compared to the number of day’s wage it would cost the country’s lowest paid unskilled worker to purchase).

### Data analysis

Data entry, validation and analysis was performed using Microsoft Excel^®^. Analysis was conducted based on facility (private/public); geographical location (North, South, Central eThekwini); and level of care (PHC, CHC, District/Provincial Hospital). All data was presented as aggregates. Raw data was stored in a password protected folder, only accessible to researchers. Medicine availability was calculated as the number of facilities stocking the listed medicine as a percentage of the total number of facilities surveyed (i.e., number of facilities stocking medicine divided by total number of facilities × 100). Affordability was assessed by calculating the cost of a treatment regimen and calculating the number of day’s wage it would cost the country’s lowest paid unskilled worker (i.e., domestic worker) to purchase that specific regimen. Accessibility was determined by assessing the above factors, together with ensuring facilities were within 1 h walk of the population.

### Quality control

Data collectors were trained in appropriate survey taking etiquette. All private sector facilities were surveyed on 1 day to avoid any major variations. Public sector facilities were surveyed within 1 month. Physical verification of stock available in the pharmacy was conducted. Before leaving the data collection site, data obtained was checked, to ensure accuracy and completeness. Data captured during data entry was double checked to avoid transfer errors.

### Ethical considerations

This study received approval from the University of KwaZulu-Natal’s Biomedical Research Ethics Committee (BE541/17), and KZN Department of Health (KZ_201805_014). Further permissions and informed consent were obtained from management at participating facilities.

## Results

### Accessibility

All selected pharmacies were accessible due to the design of the WHO/HAI facility selection criteria employed but further met the definition of accessibility as medicines were available (either on the shelf or through an order process) at these facilities and were also affordable as described further-on in this paper. Numerous pharmacies are accessible in the study location as eThekwini is the largest city in the KwaZulu-Natal (KZN) province, and most densely populated in the urban area, which was the geographical area of focus for this study. The peripheral/rural areas of eThekwini fell out of the geographical area for this study and access to priority medicines for children under 5 years would need to be evaluated in these areas in a separate study, which would be useful for comparison.

### Availability

**Fig. 1:**
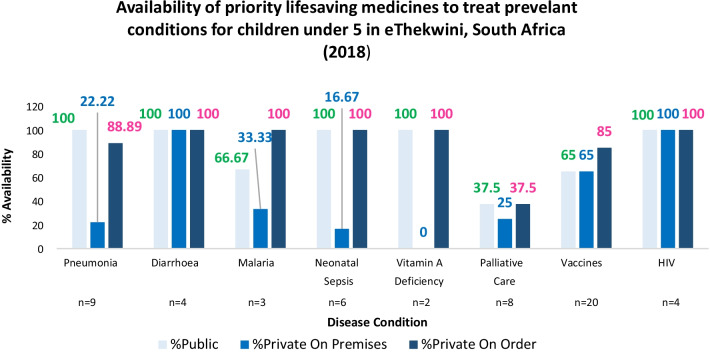
Availability of medicines for priority conditions in the private and public sector of eThekwini

Figure 1 and Additional file 1: Table S1 shows the availability of priority medicines for all eight (8) priority conditions at both public and private sector pharmacies. Overall the public sector had more medicines available on the shelf (averaging 64%) than the private sector (48%) which had more medicines available on order (84%). At least one medicine for each of the eight (8) conditions was available at both sectors which meant patients could be treated for these conditions. Medicines for diarrhoea, and HIV had the highest availability (100%) in both sectors, whilst medicines for palliative care had low availability in both sectors (37.5%). In the public sector, medicines for five (5) priority conditions (pneumonia, diarrhoea, neonatal sepsis, vitamin A deficiency and HIV) were 100% available but only medicines for two (2) conditions, diarrhoea and HIV were 100% available on the shelf for the private sector.

### Affordability

**Fig. 2:**
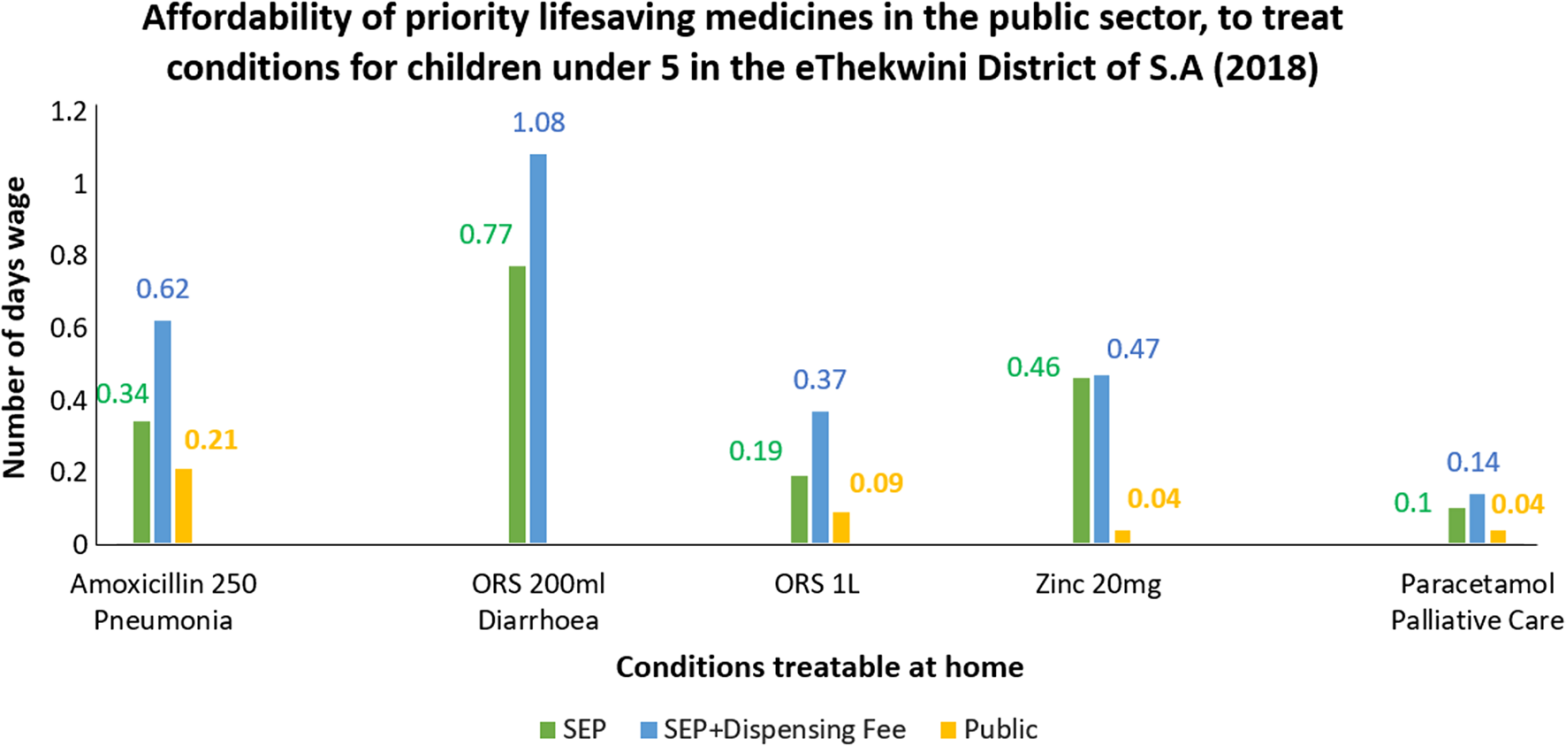
Affordability of priority medicines to treat prevalent conditions

**Fig. 3:**
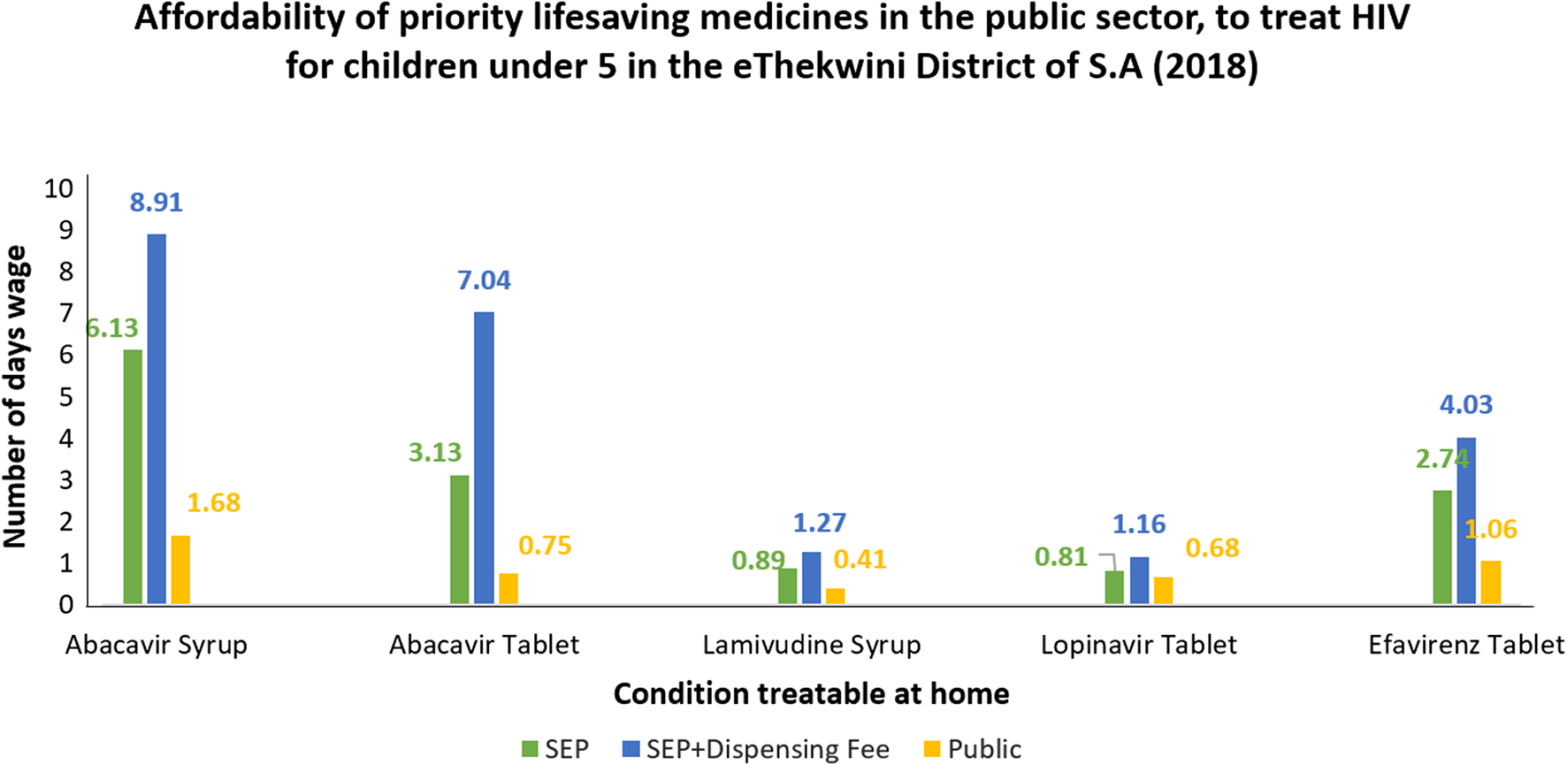
Affordability of HIV medicines

The affordability of specific medicines for the treatment of Pneumonia, Diarrhoea, Vitamin A Deficiency, Palliative care and HIV were analysed for both sectors. An average of the highest priced item from each private sector Pharmacy (SEP and SEP + Dispensing fee) was used to obtain an average that described the maximum time a person would need to work to pay for a course of treatment. To treat palliative care, pneumonia and diarrhoea in the private sector, a person would need to work 0.14, 0.37 and 0.62 days, respectively, and 9 days for a 28-day course of HIV treatment (Fig. 2). The government sector purchases medicines for the public sector. A 28-day course of HIV medicines can be obtained with a maximum of a 2-day wage in the public sector (Fig. 3). Therefore, a person would need to work less than a day to obtain a single day’s HIV treatment from both the private and public sectors. Comparing these values by wage to the private sector, it was found that to treat Palliative care, diarrhoea and Pneumonia, a person would be required to work 0.04, 0.09 and 0.21 days, respectively, in the public sector. Vitamin A was not available on the shelf in the private sector and hence the price could not be analysed.

## Discussion

Accessibility, availability, and affordability of medicines were useful indicators to assess access to medicines in eThekwini. Medicines were deemed accessible as they were within a 1-h walking distance from the surrounding populations’ residences to the nearest pharmacy [1], given that eThekwini is an urban area, medicines were also available and affordable.

Although availability of medicines on the shelf was more in the public sector than the private sector, availability of medicines per condition was deemed adequate for both sectors as availability was above 65% except for Palliative care which had low availability in both sectors (37.5%). This was possibly due to morphine granules not being available in the country although some facilities had the powder formulation. The availability of the injectable preparations in the private sector was low possibly due to these requiring a doctor’s prescription and an authorised individual to administer it. Such conditions are generally referred for hospital care. Vaccines in this study comprised of travel vaccines, as well as those on the expanded programme for immunization for South Africa (EPI-SA). Although the availability of vaccines in the public and private sector was 65% in this study, it is worth noting that the remaining 35% accounted for travel vaccines, which are available from travel clinics and private practitioners, which were not investigated in this study. Vaccines were more available on order in the private sector (85%) possibly due to difference in vaccine schedules as the public sector follows the EPI-SA, whilst the private sector includes additional vaccines not listed on the EPI-SA. Availability of vaccines may also be affected by differences in the WHO priority list being a global list and not specific for the burden of disease in SA, hence some vaccines were not necessary for the SA setting. Malaria was better catered to in the public sector (66.6%) than private sector (33.3%). Overall Malaria medicine availability was low probably due to malaria not being endemic in eThekwini and possibly not considered a priority medicine for the province; however, it is 100% available on order from the private sector, and thus access is possible. Pneumonia was adequately catered to in the public sector, 100% availability of medicines, whilst only 22.2% availability was noted on the shelf with 100% availability on order in the private sector. No exact match for dosage strength was available for Vitamin A in the private sector, only lower doses available on order and the medicine was, therefore, deemed not available for this reason. Overall the public sector had more medicines available on the shelf than the private sector which had more medicines available on order.

Although medicines for all conditions appeared to be available in private sector these medicines were available on order rather than being available on hand on the shelf at the time of the survey thus affecting actual real-time access to these medicines. This meant patients would not receive the medicines immediately and would need to wait for it to be ordered and delivered to the pharmacy potentially delaying prompt treatment. However, same day delivery is also possible if orders were placed early enough in the day but this would result in the patient incurring a second visit to the pharmacy to fetch ordered medication if a delivery service was not on offer at that specific facility. This also doubled travel costs for a patient to obtain a single treatment regimen.

The results of this study showed that the public sector in South Africa better catered to the needs of children under 5 when medicines on the shelf were considered, whilst medicines in the private sector were largely available on order. Medicines for 62.5% of the priority conditions (*n* = 8) were available in the public sector, with these pharmacies having all medicines required to treat the condition.

The affordability of the priority conditions that could be treated at home was evaluated and found to be obtainable within a 1-day, 8-h wage of the lowest paid unskilled government worker, with the exception of the HIV regimen. This meant most treatment regimens for children under 5 years were affordable.

Treatment affordability for the first line treatment of HIV did not seem affordable, although it should be noted that HIV is a chronic condition and treatment affordability was calculated based on a 28-day supply. Further analysis shows that if a daily dose for a single treatment is calculated, it would cost less than a day’s wage for both sectors. Therefore, it was deemed affordable.

Overall, medicines in the public sector were significantly cheaper than in the private sector. This is attributed to medicines in the public sector being procured via tender, based on price for volume arrangements, with the best safety, quality and efficacy profile. However, the public sector provides these medicines free of charge to pregnant woman and children, with an insignificant fee charged for those not within those categories. According to a study conducted in Malaysia in 2018 [14], it was suggested that, that the prices of essential medicines in the private sector should remain fixed to keep them at sensible levels. This can also be recommended for priority medicines for children in South Africa to ensure these medicines can be purchased at a reasonable cost and accessible to all.

Overall, it was found that all prevalent conditions were adequately catered for in both sectors in eThekwini, at affordable prices, showing that the needs of the children under 5-year population were adequately met. Priority medicines for children were more available and more affordable in the public sector.

### Study limitations

There are many private sector retail pharmacies in each area of eThekwini and it was not possible to survey all pharmacies in an area; therefore, a selected number were surveyed within a 5 km radius of the major public healthcare facility in that area. Private institutional facilities were not surveyed in this study. This is due to difficulty acquiring gate keeper permission as well as time constraints to complete the study. The system of acquiring stock was not consistent in private sector retail pharmacies. During the study it was found most retail pharmacies order medication from pharmacy wholesalers and distributors based on patients’ orders and do not necessarily keep all medicines on the shelf despite these being listed as priority by the WHO. Out of the 58 pharmacies that were meant to be surveyed, only 47% of the expected number was surveyed. This low rate limits generalisability of the study.

### Recommendations

Paediatric dosage forms were under-catered for in private sector retail pharmacies. During the study, it was found that solid dosage forms were more widely available than liquid preparations. It is speculated that this is due to the price difference between solid dosage forms and liquids dosage forms, where the liquid form is available. Therefore, policies should be put into place to ensure more child appropriate dosage forms such as syrups instead of tablets for HIV, for example, are always available at pharmacies.

More in-depth research on affordability and pricing components for medicines that treat priority conditions should be conducted. Increased awareness on the importance of accessibility, availability and affordability needs to be created amongst healthcare professionals and communities to ensure quality use of medicines. This study evaluated the urban areas of eThekwini and it is recommended this same study be conducted in rural areas of eThekwini.

## Conclusion

Priority medicines were accessible, available and affordable in the eThekwini district of South Africa. No such study for South Africa has been conducted to the knowledge of the researchers thus far. The study provides huge benefit for the province as it adds new knowledge on the access to these priority medicines for children under 5 years in eThekwini and has the potential to be up-scaled to a national study to obtain a more holistic view of access to priority medicines if conducted for each province. Such studies are important for the NHI policy makers as essential medicines feature prominently in the minimum benefits package of the NHI system.

## Supplementary Information

**Additional file 1: Table S1.** Availability of medicines at public and private sector healthcare facilities.

## Data Availability

All data generated or analysed during this study are included in this published article in the table and figures.

## Abbreviations

ACT: Artemisinin-based combination therapy
BCG: Bacille Calmette–Guerin
CHC: Community healthcare
DTP: Diphtheria, tetanus, pertussis
EML: Essential medicines lists
EPI-SA: Expanded Programme for Immunization for South Africa
HIV: Human immunodeficiency virus
HPV: Human papillomavirus
KZN: KwaZulu-Natal
NHI: National Health Insurance
ORS: Oral rehydration solution
PHC: Primary healthcare
SEP: Single exit price
UHC: Universal Health Coverage
WHO: World Health Organization
WHO/HAI: World Health Organisation/Health Action International
