# Supplementary material for: The availability of priority medicines for children under 5 years in eThekwini, South Africa
Source: J Pharm Policy Pract. 2022 Jan 5;15:2. doi: 10.1186/s40545-021-00402-y (PMC8728955; doi:10.1186/s40545-021-00402-y)
Supplement: Supplementary file 1 — Additional file 1: Table S1. Availability of medicines at public and private sector healthcare facilities. [file 40545_2021_402_MOESM1_ESM.pdf]

Supplementary Table

Table 1: Availability of medicines at public and private sector healthcare facilities

Condition	Generic Name of medicine	Dose	Dosage Form	Private Sector (%) n= 13		Public Sector (%) n= 14		
				On premises	On order	PHC n= 3	CHC n= 7	Regional n= 4
**Pneumonia**	Amoxicillin	250mg	Syrup	100	100	100	100	100
		500mg	Dispersible, scored tablet/ Flex. Solid dosage form[1]	0	0	100	66.67	100
	Ampicillin	500mg	Powder for injection	0	21.43	33.33	83.33	100
		1g	Powder for injection	0	14.29	0	16.67	25
	Ceftriaxone	250mg	Powder for injection	0	28.57	100	66.67	50
		1g	Powder for injection	0	28.57	100	100	75
	Gentamicin	40mg/ml	Injection	0	28.57	0	0	75
		20mg/ml	Injection	0	28.57	0	0	25
	Oxygen		Medicinal gas	35.71	35.71	33.33	83.33	50
**Diarrhoea**	ORS	200ml		85.71	92.86	0	0	75
		500ml		14.29	57.14	0	0	25
		1L		28.57	57.14	66.67	83.33	75
	Zinc	20mg	Syrup	28.57	71.43	100	83.33	50
**Malaria**	ACT	Optimal	Oral sol. Dose	7.14	57.14	0	16.67	25
	Artesunate	50-200mg	Injection	0	42.86	0	0	25
		50-200mg	Rectal	0	42.86	0	0	0
**Neonatal Sepsis**	Ampicillin *REPEAT*	250mg	Powder for injection	0	21.43	0	33.33	50
		500mg	Powder for injection	0	21.43	0	66.67	100
	Ceftriaxone *REPEAT*	250mg	Powder for injection	0	28.57	66.67	66.67	25

		1g	Powder for injection	0	28.57	33.33	83.33	75
	Gentamicin *REPEAT*	20mg/ml	Injection	0	28.57	0	33.33	50
	Procaine benzylpenicillin	1g	Powder for injection	7.14	28.57	0	50	50
**Vitamin A Deficiency**	Vitamin A	100000 IU		0	42.86	100	83.33	75
		200000 IU		0	42.86	100	100	75
**Palliative Care**	Morphine	20mg	Granules	0	0	0	0	0
		30mg	Granules	0	0	0	0	0
		60mg	Granules	0	0	0	0	0
		100mg	Granules	0	0	0	0	0
		200mg	Granules	0	0	0	0	0
		10mg/ml	Injection	0	7.14	100	33.33	100
		10mg/5ml	Oral Liquid	7.14	21.43	0	16.67	50
	Paracetamol		Syrup.	100	100	100	83.33	100
**Vaccines**	BCG			0	7.14	100	50	25
	Hepatitis B			21.43	62.29	33.33	83.33	50
	Polio			14.29	35.71	100	100	100
	DTP			35.71	57.14	100	100	100
	Haemophilus influenzae Type B			0	35.71	33.33	66.67	50
	Pneumococcal (conjugate)			42.86	64.29	100	100	100
	Rotavirus			42.86	71.43	100	100	100
	Measles			35.71	71.43	100	100	100
	Rubella			28.57	71.43	0	33.33	0
	HPV			35.71	64.29	33.33	83.33	50
	Japanese Encephalitis			0	0	0	0	0
	Yellow Fever			0	0	0	0	0
	Tick Borne Encephalitis			0	0	0	0	0
	Typhoid			0	7.14	0	0	0
	Cholera			0	7.14	0	0	0
	Meningococcal			14.29	57.14	0	16.67	75
	Hepatitis A			28.57	71.43	0	0	0
	Rabies			21.43	50	100	83.33	100

	Mumps			35.71	71.43	0	0	0
	Influenza (Inactivated)			57.14	85.71	33.33	0	50
**HIV**	Abacavir	Tablet or syrup		14.29	92.86	100	100	100
	Lamivudine	Syrup		28.57	92.86	50	100	100
	Lopinavir	Tablet		7.14	71.43	66.67	100	100
	Efavirenz	Tablet		14.29	71.43	100	100	100

[1]Stipulated dosage form not available as there is no 500mg syrup. Availability in the public sector depicts the availability of 500mg capsules.
